# Effect of Health Insurance on the Use and Provision of Maternal Health Services and Maternal and Neonatal Health Outcomes: A Systematic Review

**Published:** 2013-12

**Authors:** Alison B. Comfort, Lauren A. Peterson, Laurel E. Hatt

**Affiliations:** ^1^International Health Division, Abt Associates Inc., Cambridge, MA, USA; ^2^International Health Division, Abt Associates Inc., Bethesda, MD, USA

**Keywords:** Access to healthcare, Antenatal care, Facility-based deliveries, Health insurance, Maternal health, Maternity benefits, Postnatal care, Quality of service

## Abstract

Financial barriers can affect timely access to maternal health services. Health insurance can influence the use and quality of these services and potentially improve maternal and neonatal health outcomes. We conducted a systematic review of the evidence on health insurance and its effects on the use and provision of maternal health services and on maternal and neonatal health outcomes in middle- and low-income countries. Studies were identified through a literature search in key databases and consultation with experts in healthcare financing and maternal health. Twenty-nine articles met the review criteria of focusing on health insurance and its effect on the use or quality of maternal health services, or maternal and neonatal health outcomes. Sixteen studies assessed demand-side effects of insurance, eight focused on supply-side effects, and the remainder addressed both. Geographically, the studies provided evidence from sub-Saharan Africa (n=11), Asia (n=9), Latin America (n=8), and Turkey. The studies included examples from national or social insurance schemes (n=7), government-run public health insurance schemes (n=4), community-based health insurance schemes (n=11), and private insurance (n=3). Half of the studies used econometric analyses while the remaining provided descriptive statistics or qualitative results. There is relatively consistent evidence that health insurance is positively correlated with the use of maternal health services. Only four studies used methods that can establish this causal relationship. Six studies presented suggestive evidence of overprovision of caesarean sections in response to providers’ payment incentives through health insurance. Few studies focused on the relationship between health insurance and the quality of maternal health services or maternal and neonatal health outcomes. The available evidence on the quality and health outcomes is inconclusive, given the differences in measurement, contradictory findings, and statistical limitations. Consistent with economic theories, the studies identified a positive relationship between health insurance and the use of maternal health services. However, more rigorous causal methods are needed to identify the extent to which the use of these services increases among the insured. Better measurement of quality and the use of cross-country analyses would solidify the evidence on the impact of insurance on the quality of maternal health services and maternal and neonatal health outcomes.

## INTRODUCTION

Every two minutes, a woman somewhere in the world dies of pregnancy-related complications; yet, most of the deaths could be prevented using proven interventions (1). Of the 7.7 million deaths in 2010 attributed to children aged below five years, 3.1 million were in their neonatal period—within the first 28 days of life (2). Given the approaching deadline for reaching the Millennium Development Goals, the international community is encouraging low- and middle-income countries to renew their commitment to reducing maternal and child mortality rates by improving access to maternal and neonatal health services (3).

There is significant evidence demonstrating the potential effectiveness of interventions, such as access to skilled care at delivery and access to neonatal care, in reducing maternal and neonatal mortality rates respectively (4-6). However, access to these services and their quality remain low in many low- and middle-income countries. While the proportion of women who received at least one antenatal care (ANC) visit increased from 64% to 81% between 1990 and 2009 in developing countries; only 36% of women, on average, in low-income countries received the recommended four or more ANC visits between 2005 and 2010 (1). Financial barriers can play an important role in affecting timely access to maternal health (MH) services, which include ANC, skilled care at delivery, access to facility-based deliveries, and postnatal care (PNC). As a result, financial incentives, including health insurance, can address the demand-side and supply-side factors which affect the use and provision of MH services, thereby potentially influencing maternal and neonatal health outcomes.

While low- and middle-income countries are showing increasing interest in using financial incentives to encourage access to and quality of MH services, governments need evidence-based information on their effectiveness and sustainability. Consequently, the United States Agency for International Development and partners convened a Maternal Health Evidence Summit in April 2012 to bring together global experts on maternal health and health economics to present a review of the existing evidence regarding the effect of different financial mechanisms on the use and provision of MH services and maternal and neonatal health outcomes. Economists and maternal health experts assessed peer-reviewed and grey literature to answer the following questions:

1. What financial incentives, if any, are linked positively or negatively to maternal and neonatal health outcomes, the provision and use of maternal health services, or to care-seeking behaviour by women?
2. What are the contextual factors that impact the effectiveness of these financial incentives?

This paper focuses specifically on reviewing the body of evidence on insurance and its effect on the use and provision of MH services and on maternal and neonatal health outcomes. Given that a number of different financial incentives may similarly influence the provision and use of MH services, other papers in this series have focused on reviewing the body of evidence regarding their effects. These incentives include: vouchers, user fees, and conditional cash transfers (representing other demand-side financial incentives) as well as performance-based incentives (representing supply-side incentives).

This paper presents a summary of the findings from the literature taking into account both demand-side and supply-side effects and assesses the quality of the evidence. As such, the paper highlights the main conclusions that can be drawn from the literature, given the strengths and weaknesses of the existing literature. In addition, the paper discusses contextual factors which influence the effectiveness of insurance policies. Finally, the paper ends with the main conclusions drawn from the evidence and provides recommendations for both future research needs and policy tools.

### Description of the incentive

One of the main purposes of health insurance is to provide protection against financial risk. As such, health insurance can be defined as a financial mechanism that allows individuals to protect themselves against the financial cost of illness by pooling risks with others in the population (7). Insurance coverage enables individuals to replace the uncertain prospect of large financial losses with the certainty of making small, regular payments; in some cases, the payment is partially or fully subsidized by the government or a donor agency for low-income individuals.

Many middle-income and some lower-income countries offer a social health insurance scheme or national health insurance scheme. Typically, a social health insurance scheme has four features: (i) independent management of insurance funds; (ii) mandatory payroll taxes; (iii) a direct link between the contributions and the benefits package for the insured population; and (iv) a concept of solidarity (8). Often, these contributions are matched by employers or the government. In some cases, social health insurance provides access to public-sector facilities as well as approved private-sector facilities. In other cases, particularly in Latin America, the social health insurance schemes operate their own facilities, and members must use these facilities for services to be covered. National health insurance is financed through general taxation and may be mandatory for all citizens. In many cases, the government directly provides the health services (8). For the purposes of this study, schemes are identified as national versus social insurance schemes based on whether they are financed through general tax revenue or through employees’ contributions.

Governments may also offer health insurance often financed through general tax revenue and targeted at specific populations, henceforth referred to as ‘public health insurance’. Particularly in Latin America and Asia, governments may offer public health insurance schemes for low-income individuals. These schemes are often partially or fully subsidized by taxpayers or cross-subsidized by contributions of higher-income individuals to the national health insurance scheme (9). Separate public health insurance schemes may also be specifically designed for populations with a greater need for medical care and more limited resources than the average population, such as low-income pregnant women and under-five children (10).

Individuals may choose to opt for private health insurance from a commercial insurer which may enable them to obtain better benefits or often gain access to private health facilities with higher quality. Such policies are typically affordable only by wealthier groups in low- and middle-income countries. However, private micro-health insurance is also increasingly available to low-income individuals who may be excluded from schemes of the formal sector. Some micro-health insurance products are offered by large commercial insurers in partnership with local community organizations or financial institutions. These schemes often offer a very basic or subsidized benefits package to make premiums affordable. One of the main differences between private insurance policies and micro-health insurance schemes is that the former tend to be risk-adjusted, meaning that higher risk groups (such as the elderly or sicker individuals) are charged higher premiums. However, there are also examples where private micro-health insurance schemes impose risk-adjusted premiums or exclude certain groups, like individuals with pre-existing bad health conditions. Other features of these schemes may include covering transportation (such as in DEPROSC-Dhading and NIRDHAN-Banke in Nepal) (11) or offering per diems, sometimes known as “hospital cash”, during hospital stays to offset associated hospital fees and the cost of lost wages; one example is MicroFund for Women's Ri'aya product offered in Jordan (12).

Another type of micro-health insurance is community-based health insurance (CBHI) which is usually non-profit and voluntary and emerges at the community-level among those with social ties through an organization or day-to-day interaction. Group members of CBHI schemes underwrite the financial risks collectively. Mutual Health Organizations utilize similar principles as CBHI but are usually much larger and are sometimes professionally managed.

## MATERIALS AND METHODS

The reviewed literature was identified in a two-step process. First, studies published in English language over the last two decades were identified through searches conducted in key databases, using the following search terms: “maternal health outcomes”, “health insurance in low- and middle-income countries”, “maternity coverage”, “evaluation of insurance schemes”, and “health insurance coverage.” After consultation with the panel of experts, additional literature was added which had not been identified through the initial search.

The final set of articles reviewed for this synthesis included 29 articles which provide evidence on the relationship between health insurance and the use and provision of MH services as well as maternal and neonatal health outcomes. Among the original 32 studies that were identified in the literature search and by the panel of experts, 11 studies were excluded after full text review because these either did not provide evidence specifically about insurance, did not focus specifically on MH results, or provided evidence on other financing mechanisms, such as exemptions, free care, and vouchers. In addition, eight articles were added for review either because these were identified in the reference list of a reviewed article as a key source or because the authors identified these as appropriate for inclusion.

## RESULTS

### Overview of studies

Eleven of the studies provided evidence from sub-Saharan African (SSA) countries, including the Democratic Republic of Congo (DR Congo), Ghana, Mali, Mauritania, Nigeria, Rwanda, and Senegal (Table 1). Eight studies focused on low- and middle-income countries in Latin America, including Brazil, Chile, Colombia, and Peru. Nine studies used examples of health insurance from Asian countries, mostly China as well as India and the Philippines. One article studied middle-income Turkey. No reviews of cross-country evidence were included because these did not focus on the use or provision of MH service and/or did not discuss insurance.

While 16 of the reviewed studies assessed the potential demand-side effects of insurance, eight focused on the potential supply-side effects, and the remaining addressed both potential demand- and supply-side effects. Almost half of the studies used some type of econometric analysis to investigate the relationship between insurance and the outcomes of interest. Among these studies, 10 used multivariate regression analysis, two used propensity score matching (PSM), two used PSM and compared these results with results from an instrumental variable (IV) approach, one used PSM in combination with difference-in-differences (DD), one employed IV alone, and one study conducted a spatial analysis. Among the remaining studies, two tested for statistical differences in outcomes over time (without control variables), and two tested for differences in outcomes, using cross-sectional data (without control variables). Finally, seven studies presented descriptive statistics (with no statistical analysis), and one study used qualitative evidence.

**Table 1.** List of articles included in final review*

Country	Scheme	Type of scheme**	Voluntary/ mandatory	% of population covered	Study	Study design	Study focus: demand vs supply
Brazil	Government-funded “universal” health insurance and private health insurance	Public health insurance, private	Voluntary	Preventative and curative services provided to all inhabitants; 25% have PHI	Barros *et al*. (2005)	Trend analysis	Supply
					Victora *et al*. (2010)	Multivariate regression	Supply
Chile	National Insurance Fund (FONASA) Private Health Insurance	Social, private	Mandatory Voluntary	All salaried workers enrolled; 25% of healthcare users	Murray and Pradenas (1997)	Descriptive statistics	Supply
					Murray (2000)	Qualitative	Supply
China	Cooperative Medical System (CMS)	Public health insurance	Mandatory	Total population of 3,360,000	Bogg *et al*. (2002)	Trend analysis	Demand
	New Cooperative Medical Scheme (NCMS) (replaced CMS)	CBHI	Voluntary	93.37% of target population in areas offered (as of 2009)	Bogg *et al*. (2010)	Descriptive statistics	Supply
					Long *et al*. (2010)	Cross-sectional comparison	Demand
					Chen and Jin (2012)	Propensity score matching and difference-in-differences	Demand
	Employer-provided insurance (government, factory workers, farmers)	All	Mandatory (SHI), Voluntary (PHI, CBHI)	CBHI covered 80% of farmers in target population; No information provided on populations covered by govt. and labour insurance	Cai *et al*. (1998)	Multivariate regression	Supply
Colombia	“Universal” health insurance scheme where beneficiary chooses a health insurance company whose ownership may be public, private, or mixed	Public health insurance, private	Mandatory	89% of population	Giedion *et al*. (2010)	Propensity score matching and instrumental variable	Demand
Costa Rica	Mandatory insurance coverage and comprehensive primary healthcare model	Public health insurance	Mandatory	Nearly 90% of country's 4.5 million people	Cercone *et al*. (2010)	Instrumental variable	Demand
DR Congo	Bwamanda CBHI managed by district health team	CBHI	Voluntary	150,000 inhabitants of Bwamanda district	Criel *et al*. (1999)	Spatial analysis	Both
Ghana	National Health Insurance Scheme	National	Mandatory	61% of population (12.5 million people)	Chankova *et al*. (2010)	Multivariate regression and propensity score matching	Demand
					Mensah *et al*. (2010)	Propensity score matching	Demand
	Nkoranza Health Insurance Scheme	CBHI	Voluntary	33% of Nkoranza district population (44,000 people)	Smith and Sulzbach (2008)	Multivariate regression	Demand
India	ACCORD-AMS-ASHWINI community health insurance scheme, Tamil Nadu	CBHI	Voluntary	972 households (30% of all eligible households)	Devadasan *et al*. (2010)	Cross-sectional comparison	Demand
	*Yeshasvini*, Karnataka state	CBHI	Voluntary	19.5 million members	Aggarwal (2010)	Propensity score matching	Both
Mali	CBHI schemes in the district of Bla	CBHI	Voluntary	8,672 people (between 3.3 and 11.4% of the schemes’ target populations)	Smith and Sulzbach (2008)	Multivariate regression	Demand
Mauritania	Voluntary Mutual Health Organization	CBHI	Voluntary	95% of the target population in Nouakchott	Renuadin *et al*. (2007)	Descriptive statistics	Supply
Nigeria	Anambra State government-community healthcare co-financing scheme	CBHI	Voluntary	NA	Adinma *et al*. (2010)	Descriptive statistics	Both
					Adinma *et al*. (2011)	Descriptive statistics	Demand
Peru	Seguro Integral de Salud (previously Seguro Materno Infantil)	Public health insurance	Voluntary	15% of population	McQuestion and Velasquez (2006)	Multivariate regression	Both
					Bitran *et al*. (2010)	Multivariate regression	Demand
Philippines	PhilHealth	National	Mandatory for govt. and private-sector employees	Nearly 70% of all Filipinos were eligible	Kozhimannil *et al*. (2009)	Multivariate regression	Supply
					Huntington *et al*. (2012)	Descriptive statistics	Both
Rwanda	*Mutuelles de Sante*	CBHI	Voluntary	91% of the population has health insurance; Enrollment in *mutuelles* reached 85% of the target population	Hong *et al*. (2011)	Multivariate regression	Demand
					Sekabaraga *et al*. (2011)	Multivariate regression	Demand
					Lu *et al*. (2012)	Propensity score matching and instrumental variables	Demand
					Schneider and Diop (2001)	Descriptive statistics	Demand
Senegal	CBHI schemes in the Thiès region	CBHI	Voluntary	4.8% of the Thiès population (62,000 individuals)	Smith and Sulzbach (2008)	Multivariate regression	Demand
Turkey	Any health insurance	Any insurance	Voluntary	NA	Celik and Hotchkiss (2000)	Multivariate regression	Demand

*Data as of date of study;

**CBHI=Community-based Health Insurance;

NA=Not applicable;

National=National Health Insurance;

Private=Private Health Insurance;

Social=Social Health Insurance

The studies can also be categorized according to the type of health insurance scheme they evaluated. The evidence includes examples from seven national or social insurance schemes, four public health insurance schemes, 11 CBHI schemes, and three examples of private coverage. Half of the studies concentrated on CBHI schemes (which we assumed include *mutuelles,* typically found in West Africa and which are similar to CBHI but are usually larger and may employ professional management). The majority of these schemes were from SSA and/or target rural populations that are difficult to reach through traditional health insurance distribution channels. Several of these studies presented evidence from across two or more geographic locations in the same country, and one study presents evidence from three countries in SSA. As the schemes are often designed and operated at the regional or community level, policies may vary from one community to another, despite being part of the same CBHI scheme.

### Quality of evidence

The studies in this review which used econometric analyses (in half of the studies) represent the set of rigorous evidence that comes closest to establishing the causal impact of health insurance on the outcomes. One of the main challenges in identifying the causal effect of health insurance is being able to control for the endogeneity or selection bias in insurance uptake. Individuals who enroll in insurance tend to be different, on average, from uninsured individuals. This is a consistent challenge, especially in contexts where wealthier individuals are both more likely to enroll in insurance and more likely to use MH services. Alternatively, sicker individuals may be more likely to enroll in insurance as well as be more likely inclined to use health services, resulting in adverse selection. Both these selection effects could explain a positive relationship between health insurance and the use of MH services rather than demonstrate the causal effect of insurance.

A randomized controlled trial design is the only method that can eliminate this endogeneity because the treatment and control groups should be similar, on average, both for observable and unobservable characteristics. However, none of the studies in the review used this methodology. Some of the strongest studies attempted to control for endogeneity by using PSM (13-17) where they compared insured and uninsured individuals by matching them on observable characteristics. However, this method assumes that, when matched on observables, individuals should be similar in unobservable characteristics (18). These studies matched on age, marital status, education, and asset ownership; however, unobservable characteristics, like baseline health status, could also differ between the insured and the uninsured. Only one of the studies using PSM included baseline health status as a matching variable as well as distance to health facility (13).

Two studies conducted cross-sectional regressions to compare outcomes between geographical areas where insurance was introduced and control areas without insurance (19,20). One of the concerns is that characteristics at the district-level may differ and be correlated with the introduction of insurance, which would bias the results. Another set of studies used multivariate regression analyses to compare insured and uninsured individuals (10,21-27). Omitted variable bias remains a concern here since these studies may not have controlled for all characteristics that may differ between insured and uninsured individuals. Only one of the reviewed studies used both PSM and DD to account for both the endogeneity of insurance uptake and differences in county-specific unobservables (14). However, this study did not use DD as a way to assess changes in outcomes over time due to insurance. No study in the review used DD for that purpose. The three studies which used an IV approach provided limited information on the instrument, making it difficult to assess whether it provides an independent source of variation for insurance uptake (16,17,28).

The remaining set of studies, which conducted simple statistical tests either on an outcome over time or on the difference in the outcome between insured and uninsured individuals, cannot provide rigorous evidence for the impact of insurance, largely because of omitted variable bias (29-32). There are numerous potential confounders which could explain differences in outcomes between the insured and the uninsured. Moreover, any changes in outcomes over time could be wrongly attributed to the introduction of insurance because of other changes that may also be occurring at the same time. The studies that provided descriptive statistics cannot be used for causal inference about the effect of insurance. Finally, the qualitative evidence can be used for obtaining a more nuanced understanding of the effect of insurance and complement quantitative data.

### Potential pathways for the effect of insurance

There are various pathways through which insurance may ultimately affect maternal and neonatal health outcomes (Figure). First, insurance may influence the use of MH services through the reduction in the price. In turn, greater use of MH services, which are known to influence MH outcomes, should reduce maternal mortality and other related health outcomes. In addition, insurance may influence the quality of MH services through provider accreditation processes, modes of provider payment, and, more generally, by ensuring consistent flows of funding to providers. If women are accessing MH services and if these services are of poor quality, the expected health benefits related to the use of MH services may not occur. This review assesses the evidence based on these different pathways.

### Summary of findings

#### Effects of health insurance on the use of MH services

None of the studies can conclusively demonstrate a causal relationship between insurance and maternal healthcare-use because none relied on randomized methods. The literature does consistently indicate the expected associations between insurance and MH service-use. Among the studies which focused on facility-based deliveries and skilled attendance at birth, there was mostly consistent evidence that health insurance is positively correlated with both measures. The studies provided examples of this positive correlation in different geographic areas, including SSA (Ghana, Mali, Rwanda, and Senegal), Asia (India and China), Latin America (Peru and Colombia), and Europe (Turkey).

**Figure.**
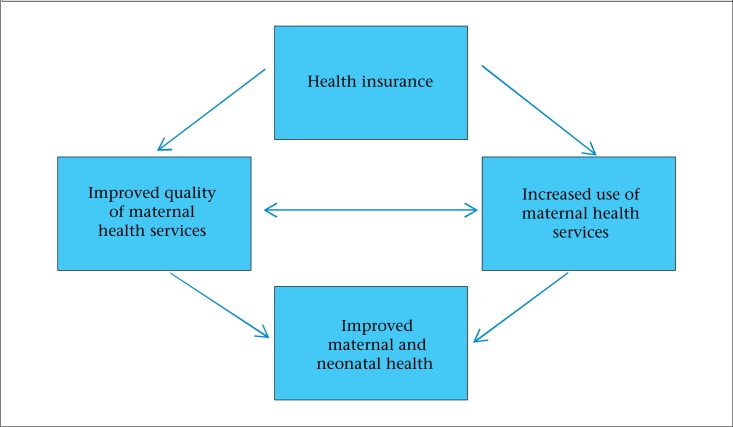
Pathways for effect of insurance on the use of MH service, quality, and health outcomes

The association between insurance and facility-based deliveries and skilled attendance at birth was also consistent across different types of insurance schemes. One example of a national health insurance scheme came from China where 100% of women in the sample who were covered by social health insurance scheme had skilled attendance at delivery compared to 91% of enrollees in the rural Co-operative Medical Systems (CMS) and 46% of uninsured women (30). There is conflicting evidence from the National Health Insurance Scheme (NHIS) in Ghana: one study found that NHIS members were significantly more likely to deliver in a hospital and deliver with professional assistance (15) whereas another study failed to find such an effect, using multivariate regression analysis on pre-post data (23). Evidence from public health insurance schemes came both from Colombia for the subsidized national health insurance schemes targeted at the poor (17) and from Peru for the Maternal and Child Health Insurance (SMI) programme. In Peru, women who were eligible for SMI had twice the odds of delivering in a facility compared to ineligible women (20). In contrast, another study which looked at the public health insurance programme in Peru once it was scaled-up nationwide found no detectable effects on deliveries with skilled attendance (10). The discrepancy between the findings could be due to factors, such as quality of the programme after scale-up and the quality of available providers. Alternatively, the initial implementation phase of SMI, which the first study assessed, may have occurred in areas with poorer baseline outcomes.

A number of studies also provided evidence from CBHI schemes. For example, in Rwanda, CBHI members were 1.6 times more likely to deliver in a modern health facility compared to uninsured women (25). Another study from Rwanda's CBHI scheme found that insured women are three times more likely to deliver with professional assistance compared to uninsured women who are more likely to deliver at home alone (33). Similar evidence exists for studies of CBHI schemes in Nigeria (34,35), Mali and Senegal (26), and India (31). However, evidence from another study in India on the *Yeshasvini* health insurance scheme in the state of Karnataka found no detectable effect on facility-based deliveries (13). The authors explain the absence of an effect as likely due to deliveries being free of charge in public facilities. This particular insurance, therefore, benefits enrollees through their increased access to private providers rather than through cost reductions. For comparison purposes, the other studies do not provide information on the cost of facility-based deliveries at non-affiliated providers. Another factor that could explain the absence of detectable effects is related to the baseline average for these variables; an effect will be more difficult to detect if outcome levels are very low or very high. Aggarwal (2010) does not provide baseline estimates for facility-based deliveries (13). Among the other studies, there is significant variation in terms of baseline estimates; in Mali and Senegal, the rates of facility-based deliveries are 65% and 71% respectively (26) compared to 27% in Rwanda (25) (Table 2 for baseline outcomes and effect-sizes).

Most of the studies found a consistently positive relationship between health insurance and both probability of women using any ANC and the probability of women receiving at least four ANC visits during their pregnancy. The exceptions include a study from China on the New Co-operative Medical Systems (NCMS) (32) and a study from India on the *Yeshasvini* CBHI scheme (13) which found no detectable effect. One reason for the absence of an effect in China is the fact that, in all counties, only 1% or less of women did not have prenatal care visits at baseline, making it unlikely to detect an effect; no baseline outcome variables are presented for the *Yeshasvini* scheme (13) (Table 2). In Senegal and Ghana, there were also no detectable effects of CBHI coverage on receiving at least four ANC visits or receiving ANC during the first trimester (26). Another study in Ghana found that NHIS coverage did not affect the use of ANC (23). Examples for the positive relationship between insurance and ANC visits came from Mali (26), Nigeria (34,35), Rwanda (33), Ghana (15), China (30), Colombia (17), and Turkey (22). Similarly, these studies cover examples of national health insurance schemes (15), CBHI schemes (26,33-35), and public health insurance schemes (17,30). Among the studies which reported results for PNC, there was also a consistently positive relationship between health insurance and the use of postnatal care. Examples of this relationship came from studies in Ghana (15), Mauritania (36), and China (30). However, only Mensah *et al.* (2010) used rigorous evaluation methods to identify this relationship, through PSM (15).

The majority of these studies which focused on the relationship between insurance and the use of MH services assumed that the main pathway for the effect of insurance was through the reduction in the costs associated with seeking care. However, two studies focused on an alternative pathway through the effect of insurance on the quality of providers (19,37). The example came from the Philippines where the National Health Insurance Program administered by PhilHealth required the simultaneous accreditation of public and private healthcare institutions at all levels. Kozhimannil *et al*. (2009) found that an increase in the number of PhilHealth facilities per 10,000 births was associated with a significant increase in the probability of receiving four ANC visits during pregnancy and an increase in the probability of receiving ANC visits during the first trimester; no significant effect was identified for deliveries in health facilities (19). These effects suggest that access to higher-quality facilities, namely facilities that meet a minimum of standards set by the government, is related to greater use of certain MH services.

The most rigorous studies among those that focus on the use of MH services relied on PSM (13,15,16), one of which also used an IV approach (17). While an IV approach can provide rigorous evidence of a causal effect, this study did not present any information about the instrument used, thereby making it difficult to evaluate its validity. Aggarwal (2010) presents the strongest example of likely causality in their study of the *Yeshasvini* CBHI scheme, given that the matching includes not only demographic and socioeconomic variables but also health status measured by the presence of chronic disease and distance to health facility (13). Nonetheless, this study found no detectable effect from insurance on the use of MH services. This suggests that, while most of the evidences demonstrate a positive correlation, there is a need for robust evidence to establish causal inference, given that the most rigorous studies find no effect.

Overall, the evidence shows that health insurance is correlated with greater access to key services, such as ANC, facility-based deliveries, deliveries with SBAs, and PNC, particularly in settings where access to these services is low at baseline. The findings from these studies are consistent with economic theory which predicts that generous insurance coverage (through lower co-insurance rates and lower deductibles) lowers the cost of healthcare to consumers and, thus, will lead to higher use of healthcare (38).

#### Effects of health insurance on the provision of MH services, including quality of care

Health insurance could influence the volume and quality of MH services provided by affecting providers’ behaviour. In some instances, increased service volumes are a desirable outcome while, in other instances, overprovision may be a concern. Among the studies which focused on the potential effect of insurance on the provision of MH services, many provided suggestive evidence of overprovision of caesarean sections (C-sections), possibly due to supplier-induced demand. This occurs when the provider influences a patient's demand for care against the provider's own interpretation of the patient's best interest (39). In the context of MH services, supplier-induced demand may occur if the provider recommends a C-section when it is not medically necessary. Patients may also request C-sections for convenience or other cultural reasons.

**Table 2.** Summary of baseline outcomes and effect-sizes by study

Country	Scheme(s)	Study	Baseline indicator (prior to insurance or among control group)	Effect identified by study among insured/at follow-up†
Brazil	- Government-funded “universal” health insurance - Private health insurance	Barros *et al*. (2005)	Outcomes at baseline (pre-insurance-1982): - 61.5% ANC in first trimester - 61% of deliveries with medical doctor - 28% C-section rate	Effect at follow-up (post-insurance-2004) - 10.8 ppt increase in ANC visits in first trimester (p=0.0001) - 27.7 ppt increase in attendance by medical doctor (p=0.0001) - 15.0 ppt increase in C-section rate (p=0.0001) - 22% reduction in birthweight-specific neonatal mortality rates (1982 to 2004) - 49% reduction in gestational-age specific neonatal morality (1982 to 2004)
		Victora *et al.* (2010)	Outcomes among publicly-insured (ANC tests): - 97.5% blood analysis - 96.5% urine analysis - 75.9% iron supplement prescription - 22.2% vitamin prescription - 97.4% ultrasound - 99.3% uterine height measurement - 99.8% blood pressure measurement	Effect among privately insured: - 2.5 ppt higher probability blood analysis (p<0.001) - 3.0 ppt higher probability urine analysis (p<0.001) - 5.3 ppt higher probability iron supplement prescription (p<0.001) - 26.4 ppt higher probability vitamin prescription (p<0.001) - 2.1 ppt higher probability ultrasounds (p<0.001) - No effect on uterine height measurement - No effect on blood pressure measurement
Chile	- National Insurance Fund (FONASA) - Private health insurance	Murray and Pradenas (1997)	Outcomes at baseline (pre-insurance): - 27.7% C-section rate (1986)	Effect at follow-up (post-insurance): - 9.5 ppt increase in C-section rate (1994) No statistical tests conducted
		Murray (2000)	Qualitative data	Not applicable
China	- Insurance for government employees - Insurance for labourers, - Cooperative Medical Scheme (CMS)	Bogg *et al*. (2002)	Outcomes among uninsured: - 55% ANC - 31% 3+ ANC visits - 40% facility-based delivery - 46% delivered with SBA - 26% PNC (1975-1979)	Effect among insured: - 30 ppt higher probability of ANC (government or employer insurance) and 28 ppt higher probability of ANC (CMS) (p<0.001) - 49 ppt higher probability of 3+ ANC visits (government or employer insurance) and 40 ppt higher probability of 3+ ANC (CMS) (p<0.001) - 49 ppt higher probability facility-based delivery (government or employer insurance) and 47 ppt higher probability of facility-based delivery (CMS) (p<0.0001) - 54.4 ppt higher probability of SBA (government or employer insurance) and 45 ppt higher probability SBA (CMS) (p<0.0001) - 21 ppt higher probability of PNC (1990-1995) (p<0.0001) - Women paying out of pocket compared to insured women were 4.54 times more likely to experience adverse pregnancy outcomes (p<0.01)
	New Cooperative Medical Scheme (NCMS) (replaced CMS)	Bogg *et al*. (2010)	Outcome in five counties at baseline (2004): - 8.3%, 12.2%, 32.2%, 35.0%, 60.0%	Effect in counties at follow-up (2007) - 19.2%, 18.6%, 439.0%, 56.4%, 60.0% C-section rates No statistical tests conducted
		Long *et al*. (2010)	Outcomes in counties where ANC is not covered: - 99% ANC - 75% and 75% ANC in first trimester	Effect in counties where ANC is covered: - No effect on ANC - No effect on ANC in first trimester
		Chen and Jin (2012)	Outcomes in non-NCMS counties: - 2.3% pregnancy related deaths	Effect in NCMS counties: - No effect on pregnancy-related deaths
	Employer-provided insurance (government, factory workers, farmers)	Cai *et al*. (1998)	Outcomes among women with cooperative insurance (reference group) - 10.8% C-section rate	Effect among other insurance groups: - Women with government insurance are 5.77 times more likely to deliver by C-section (p<0.05) - Women with social insurance were 3.31 times more likely to deliver by C-section (p<0.05) - No difference for rural social insurance
Colombia	“Universal” health insurance scheme where beneficiary chooses a health insurance company whose ownership may be public, private, or mixed	Giedion *et al*. (2010)	Outcomes among uninsured: - 80.8% delivered with SBA - 81.2% facility-based deliveries - 5.3 ANC visits - 32.2% complication after delivery - 5.2% low birthweight	Effect among insured: - 7 ppt higher probability of SBA (p<0.01) - 7 ppt higher probability of facility-based delivery (p<0.01) - 6 ppt increase in number of ANC visits (p<0.01) - No effect on complications after delivery - 63 ppt increase probability of low-birthweight (p<0.01)
Costa Rica	Mandatory insurance coverage and comprehensive primary care model	Cercone *et al*. (2010)	No details for uninsured	- Results not shown; analysis finds that insured women are less likely to have low-birthweight babies
DR Congo	Bwamanda CBHI managed by district health team	Criel *et al*. (1999)	Outcome among uninsured: - 0.74% C-section rate	Effect among insured: - 1.97% C-section rate No statistical test conducted
Ghana	National Health Insurance Scheme (NHIS)	Chankova *et al*. (2010)	No baseline data provided	Effect at follow-up (after insurance roll-out): - No effect on 4+ ANC visits - No effect on facility-based deliveries - No effect on delivery by C-section
		Mensah *et al*. (2010)	Outcomes among uninsured: - 72% ANC - 59% 3+ ANC visits - 78% weight measurement - 79% blood pressure test - 66% blood testing - 69% urine testing - 9.4% infant death - 7.5% birth complications - 47% delivered with SBA - 53% facility-based delivery - 71% PNC	Effect among insured: - 13.8 ppt higher probability of ANC (p<0.01) - 22.8 ppt higher probability of 3+ ANC visits (p<0.01) - 15 ppt higher probability of weight measurement (p<0.01) - No effect on blood pressure test - No effect on probability of blood testing - No effect on probability of urine testing - 8 ppt lower probability of infant death (p<0.01) - 3.3 ppt lower probability of birth complications (no statistically significant but other specifications are) - 15 ppt higher probability of delivery with SBA (p<0.05) - 17 ppt higher probability of facility-based delivery (p<0.01) - 16 ppt higher probability of PNC (p<0.01)
Ghana, Mali, Senegal	- Nkoranza Health Insurance Scheme (Ghana) - CBHI schemes in district of Bla (Mali) - CBHI schemes in the Thiés region	Smith and Sulzbach (2008)	Outcomes among uninsured: Ghana: - 79% 4+ ANC visits - 65% facility-based delivery Mali: - 35% ANC in first trimester - 25% 4+ ANC visits - 65% facility-based delivery Senegal: - 72% ANC in first trimester - 54% 4+ ANC visits - 71% facility-based delivery	Effect among insured: Ghana: - No effect on 4+ ANC visits - No effect on facility-based deliveries Mali: - Insured are 2.37 times more likely to receive ANC in first trimester (p<0.05) - Insured are 2.41 times more likely to received 4+ ANC visits (p<0.05) - Insured have 4.32 times higher probability of facility-based deliveries (p<0.10) Senegal: - No effect on ANC in first trimester - No effect on 4+ ANC visits - Insured have 4.74 times higher probability of facility-based deliveries (p<0.10)
India	*Yeshasvini*, Karnataka state	Aggarwal (2010)	No baseline data provided	- No effect on facility-based deliveries - No effect on ANC visits - 5.4 ppt lower probability of C-section among uninsured (p<0.05)
India	ACCORD-AMS-ASWINI (AAA) CBHI scheme, Tamil Nadu	Devadasan *et al*. (2010)	Outcomes among uninsured: - 45% delivered in a hospital	Effect among insured: - 45.0 ppt higher probability of facility-based delivery (p<0.01)
Mauritania	Voluntary Mutual Health Organization	Renaudin *et al*. (2007)	Outcomes at baseline (at insured facilities-2003): - 80% filled in partograph - 41% PNC	Effect at follow-up (at insured facilities-2005): - 33 ppt decrease probability of partograph filled - 28 ppt increase in probability of PNC
Nigeria	Anambra State government-community healthcare co-financing scheme	Adinma *et al*. (2010)	Outcomes at baseline (early intervention-2004) - 72 women received ANC - 33 women had facility-based deliveries	Effect at follow-up (2005) - 129 women received ANC (p<0.05) - 74 women had facility-based deliveries (p<0.05)
		Adinma *et al*. (2011)	Same data as above, with control group	Same data as above No statistical tests conducted
**Contd.**				
Peru	Seguro Integral de Salud (previously Seguro Materno Infantil)	McQuestion and Velasquez (2006)	No baseline data provided	- Women insured by SMI are 2.02 times more likely to have facility-based delivery (p<0.05)
		Bitran *et al*. (2010)	No baseline data provided	- Analysis results not shown; analysis finds no effect from insurance on probability of delivery with SBA
Philippines	PhilHealth	Kozhimannil *et al*. (2009)	Outcome at baseline (pre-intervention): - 55% ANC in first trimester - 64% 4+ ANC visits - 37% facility-based deliveries	Effect in catchment area with facility improvements: - 3 ppt increased probability of ANC in first trimester p<0.05) - 4 ppt increased probability of 4+ ANC visits (p<0.05) - No detectable effect on facility-based deliveries
		Huntington *et al*. (2012)	Outcome at baseline (pre-intervention) - 254 maternal deaths per 100,000 livebirths	Effect at follow-up: - MMR decreases; 114 maternal deaths per 100,000 livebirths (*no statistical tests conducted)*
Rwanda	*Mutuelles de Sante*	Hong *et al*. (2011)	Outcomes among uninsured: - 74% home delivery - 65% delivered without the assistance of a skilled birth attendant	Effect among insured: - 29 ppt lower probability of home delivery (p=0.000) - 25 ppt lower probability of delivery with unskilled birth attendant/unassisted (p=0.000)
		Sekabaraga *et al*. (2011)	Outcomes among entire sample (insured and uninsured): No baseline data on facility-based deliveries - 27% SBA	Effect among insured: - 1.6 times more likely to have facility-based delivery (p=0.0000)
		Lu *et al*. (2012)	Outcomes among uninsured: - 59.5% SBA (among uninsured post-intervention) - 1,071 maternal death per 100,000 livebirths	Effect among insured: - Insured women are 2.3 times more likely to have SBA (p=0.000) - MMR decreases; 540 maternal deaths per 100,000 livebirths (*no statistical test conducted*)
	Prepayment scheme	Schneider and Diop (2001)	Outcomes among uninsured: - 83% 1+ ANC visits - 40% 3+ ANC visits	Effect among insured: - 7.0 ppt higher probability of 1+ ANC visits - 14.0 ppt higher probability of 3+ ANC visits - Insured women are three times more likely to have facility-based delivery (*no statistical tests conducted)*
Turkey	Any health insurance	Celik and Hotchkiss (2000)	No baseline data provided	Effect among insured: - Insured women are 1.79 times more likely to have received ANC (p<0.01)

†The effect identified depends on methodology of the study. Either it represents the change over time in the indicator, for longitudinal data or for pre-post comparisons among insured groups, or it represents the effect of insurance among the insured compared to the uninsured

### Volume of C-sections provided

Studies which find a positive relationship between health insurance and C-sections are not necessarily identifying supplier-induced demand since this positive relationship could be the result of previously high unmet need for C-sections or result from clients’ demand for C-sections. Seven studies in the review found a positive relationship between health insurance and the rate of C-sections. The evidence primarily came from Latin America (Brazil and Chile) and Asia (China and India). Evidence from Latin America focused on private health insurance (29,40,41). Descriptive trend data from Chile showed that the rate of C-sections increased by one-third during the same period that the proportion of women covered by private insurance increased substantially (40). Qualitative data from Chile suggest that physicians preferred serving private maternity patients because of the higher financial remuneration (41). In China, one study found that government employees with government insurance were five times more likely to deliver by C-section, and women with social health insurance were three times more likely to deliver by C-section compared to women with cooperative insurance. There was no difference in C-section rates among women with social health insurance in rural areas, women with cooperative insurance, or women who self-paid (21). Evidence from the implementation of NCMS in rural China, an example of CBHI insurance, showed a substantial increase in C-section rates associated with the introduction of this insurance policy (42). The study of the *Yeshasvini* CBHI scheme in India, however, found that membership did not increase the number of C-sections and that, among lower-income households, C-section rates were 30% lower than in uninsured low-income households (13). In Ghana, higher rates of C-sections were found among the insured NHIS population (23).

Although some studies demonstrate a positive correlation between insurance and the provision of C-sections, none of the studies conclusively proves that supplier-induced demand is occurring. It is difficult to demonstrate that the rate of observed C-sections is not medically necessary. A related point is that there may exist adverse selection; insured women may represent higher-risk women who have a greater need for C-sections.

Nonetheless, the population-level estimates of C-section rates presented in many of these studies are suggestive of overprovision, particularly in Latin America and in China. For example, Barros *et al*. (2005) show that C-section rates increased from 28% in 1982 to 31% in 1993 and 43% in 2003 for representative cohorts of women in urban Brazil (29). Murray and Pradenas (1997) present national-level estimates of C-section rates in Chile, which increased from 27.7% in 1986 to 37.2% in 1994 (40). In rural China, county-level estimates for C-section rates in five counties show increases, over three years, from 8.3% to 19.2% in one county, from 12.2% to 18.6% in the second, 32.2% to 43.9% in the third, 35.0% to 56.4% in the fourth, with no increase in the fifth county (42). Each of these studies shows examples of countries that exceeded the WHO-recommended maximum expected rate of C-sections of 15% (43). Overall, this evidence is consistent with studies that have identified the rate of C-sections by geographic region (44) as well as identified which countries over- and underprovide C-sections (45); this recent study finds that 40% of the countries fall beneath the 10% rate of C-sections, considered to be the threshold for underprovision, and the majority of these countries are in sub-Saharan Africa. In contrast, 50% of the countries fall above the 15% threshold, considered to be the threshold of overprovision; China and Brazil account for the majority of overprovided C-sections (45).

Some of the studies suggested pathways through which insurance could explain potential supplier-induced demand. Cai *et al.* (1998) noted that physicians’ payment switched from salary-based to quasi-fee for-service through which they received higher payments for C-sections (21). In addition, physicians’ bonuses were linked to hospital revenue, where hospitals are reimbursed more for longer lengths of stays (21,42). In contrast, the evidence from India suggested that the schemes set below market rates for the reimbursement of C-sections, which led providers to avoid patients potentially requiring C-sections (13). Most of these studies presented descriptive statistics, including trend data and qualitative data. Only two of the studies used PSM (13,15). Aggarwal *et al*. (2010) uses comprehensive matching variables, including health status proxied by the presence of chronic illness and was the one to identify a lower rate of C-sections among low-income insured individuals (13). Overall, more rigorous research is necessary to effectively demonstrate whether the observed relationship between insurance and C-section rates in these low- and middle-income contexts is due fully or in part to supplier-induced demand. It could also be the case that insurance influences women's requests for C-sections to avoid vaginal deliveries, particularly if they do not bear the full cost through insurance; the one-child policy in China has been cited as a reason for women desiring C-sections (21).

### Quality of MH services

Four studies focused on the potential effect of insurance on the quality of MH services, and these studies varied significantly in terms of how they measured quality, making it difficult to compare results across studies. Theoretically, health insurance could affect the quality of MH services through various pathways, including accreditation requirements for reimbursement by insurers, providers competing on quality to attract insured patients, or greater revenue generation at the facility-level which enables providers to invest in quality improvements. The evidence was inconsistent with regard to the relationship between health insurance and the quality of MH services.

Two studies focused on the types of tests conducted during ANC visits as a measure of quality. In Ghana, members of NHIS were more likely to have their weight and blood pressure measured but there was no significant effect on insurance enrollment on urine or blood testing, and contradictory findings for blood pressure testing (15). In Brazil, privately-insured patients were more likely than patients insured through the national insurance scheme to have blood testing, urine testing, ultrasounds, and prescriptions for vitamins and iron. However, they were not more likely to have uterine height or blood pressure measured (27). A study from Mauritania measured quality based on whether the partograph was filled in and done correctly based on a review of delivery records at facilities covered by insurance; this study found a decrease, over time, in the percentage of deliveries with a partograph filled in (36). Another study measured quality by the type of health personnel present at delivery; this study found that, following the introduction of national health insurance programme, a larger proportion of births occurred with a medical doctor in charge compared to a nurse or medical student, and a larger portion of births occurred with a paediatrician in the delivery room (29).

Some of the studies suggested possible explanations for the observed quality changes. For example, in Mauritania, the observed reduction in quality was explained as being related to increased workload for the direct service providers as a result of more insured patients being seen while providers’ pay remained constant (36). Adinma *et al*. (2010) suggested that quality improvements relating to the introduction of insurance, in turn, motivated providers to improve delivery of services in Nigeria (35).

Overall, the evidence on the relationship between health insurance and the quality of MH service provision is inconclusive because of the differences across studies in the quality measures used, variation in the direction of the relationship, and reliance on descriptive methods. Measuring the quality of maternal healthcare is generally problematic and, to date, there is no consensus among maternal health experts on the best quality care measures. Only one study used multivariate regression analysis (27); another used PSM but did not match on variables, such as health status (15). Therefore, this review was unable to identify evidence of a causal relationship between insurance and the quality of MH service provision.

#### Effects of health insurance on maternal and neonatal health outcomes

There is little evidence available about the relationship between health insurance and maternal or neonatal mortality because few studies have measured these outcomes. Among the three studies that focused on insurance and maternal mortality, only one was rigorously conducted. That study found no detectable effect from enrollment in NCMS in China on pregnancy-related deaths, although the study was not likely powered to detect such effects (14). This was the only study in the review to have used both PSM and DD, thereby controlling both for endogenous insurance enrollment and differences in insurance availability by geographic area. The other two studies both identified decreases over time in the maternal mortality ratio as insurance coverage increased; however, it was not possible to disentangle the role of insurance compared to other concurrent sector-wide reforms or interventions (16,37).

The available evidence (in two studies) identified a negative correlation between health insurance and neonatal deaths (15,29). In Brazil, gestational age-specific neonatal mortality and birthweight-specific neonatal mortality decreased among birth cohorts over time as insurance coverage expanded. However, it is not possible to disentangle the contribution of other factors, such as concurrent quality improvements over time unrelated to the changes in insurance policy. Another study, which focused on miscarriages and stillbirths, found that women paying out of pocket were 4.54 times more likely to experience these adverse pregnancy outcomes compared to insured women in China (30). This study presented trend data, without controlling for potential confounders.

There is conflicting evidence on the relationship between health insurance and birth complications; one study in Ghana finds that, for certain specifications in the analysis, NHIS members are less likely to have birth complications (15) whereas a study in Colombia finds no detectable difference in birth complications between those insured in the public health insurance scheme and uninsured women (17). While both studies used PSM, there is either limited information about the matching variables used or insufficient matching variables.

Similarly, the evidence available for the relationship between health insurance and birthweight is also contradictory. Insured women in Costa Rica had a lower probability of having a baby with low birthweight (28). In contrast, insured women in Colombia were more likely to have a baby with low birthweight; the authors do not explain this finding (17). While both of these studies used IV, neither provides sufficient information about the instrument used. Over time, as insurance coverage expanded, birth cohorts in Brazil were found to have lower birthweight and lower gestational age at birth (29). These results are based on descriptive trend data, with no analysis of potential confounding factors.

Most studies which identified a relationship between health insurance and these health outcomes also provided suggestive evidence that this relationship was mediated through the effect of insurance on access to care. For example, NHIS members in Ghana, who were less likely to experience infant deaths or have birth complications, were more likely to have ANC visits and more likely to have more intensive testing during these visits (15). In contrast, the decrease over time in birthweight observed in Brazil, despite increases in insurance coverage, was attributed to greater use of medical technology, including ultrasound, which may have resulted in inducing labour or performing C-sections earlier (29). In the China example, the authors suggested that the absence of an effect on maternal mortality is linked to low reimbursement rates to cover enrollees’ costs which would limit their use of health services. Quality of MH services may also play a role in influencing these health outcomes; one study from Brazil mentioned that the low birthweight could be related to the poor quality of ANC services, including drug availability (29).

Overall, the evidence regarding the relationship between health insurance and maternal and neonatal health outcomes is inconclusive, partly due to the smaller number of studies focusing on these outcomes, in addition to conflicting findings among the studies. While some of the studies use rigorous evaluation methods, including PSM and IV, the articles provide insufficient information about their methodologies, making it difficult to evaluate these approaches. Only one study provides a comprehensive description of the methodology, using both PSM and DD; yet, this study fails to identify a relationship between health insurance and maternal mortality.

#### Contextual factors

The effect of insurance on MH service-use and provision as well as on maternal and neonatal outcomes may be influenced by contextual factors. We draw from the literature the main examples of important contextual factors.

### Geographic access to providers

One of the important contextual factors which may influence the effect of insurance on the use of MH services is the accessibility of providers within the network of covered providers. Particularly in rural locations, access to providers may be an important constraint that limits the ability of insured populations to seek and receive care when needed. In addition, the transportation costs (including both direct costs and the opportunity cost of time for seeking care far from home) may represent significant costs, especially to low-income individuals. Most insurance policies do not include transportation costs as part of their benefits package. The reviewed studies provide conflicting evidence regarding whether health insurance can overcome geographic barriers to care. In DR Congo, there was no difference in the rate of C-sections among the insured population, regardless of individuals’ residential distance to facility; in contrast, the rate of C-sections was lower among uninsured individuals who lived further from the facility (46). In contrast, a study in India found that, as distance from the hospital increased, utilization of hospital services decreased regardless of insurance status (31). While there was no mention of transportation benefits included in the insurance coverage in DR Congo, transportation was not included as a benefit in the example from India.

### Quality of care

The extent to which insurance provides access to better-quality care might influence the magnitude of its effect on demand for MH services as well as its effect on maternal and neonatal outcomes. If insurance enables individuals to access services from higher-quality providers because these providers are included in the network of covered providers, there may be an overall increase in demand for MH services as they are more highly valued; in turn, the use of higher-quality MH services could positively affect health outcomes. Reductions in neonatal mortality in Brazil may have been partly due to insurance-related quality improvements over time, including the availability of more trained staff, increased bed-capacity, universal access to surfactants, and better respirators and lab techniques (29). In contrast, the evaluation of Proyecto 2000 in Peru, which involved improvements in physical infrastructure, improvements in the quality of care provided, and expansion of social health insurance coverage found that women who lived in areas where quality improvements were implemented were not more likely to deliver in a health facility (20). However, the authors attributed this to a potential lack of awareness among the target population about these facility improvements.

#### Sustainability

There was limited mention of the sustainability of the insurance schemes reviewed in this analysis. Adverse selection (when higher-risk individuals have a higher probability of enrollment) and moral hazard (when insured individuals utilize more services than they would if they were bearing the full cost) are classic threats to the financial viability of insurance. One example of adverse selection occurred in the Obstetric Risk Insurance scheme in Mauritania, a voluntary insurance scheme intended only for pregnant women with no waiting period (36). Since the insurance only provides maternity benefits, there is no broader risk-sharing with non-pregnant women who may incur less costs for the insurer, which would potentially threaten the long-term financial sustainability of this scheme. In Ghana, Chankova *et al*. (2008) found that higher-income women who had a delivery in the last year were more likely to enroll in NHIS compared to women who did not have a delivery (23). The authors posited that this higher rate of enrollment was socially beneficial; nonetheless, it has important implications for the financial sustainability of the scheme.

The high rates of C-sections in Brazil, India, and China discussed earlier could indicate moral hazard if women opted for a C-section rather than a vaginal delivery because it was fully covered by insurance. In addition to raising concerns about putting women unnecessarily at risk, high rates of C-sections might not be financially sustainable for government or private insurers. However, the studies did not address whether overprovision of C-sections was associated with sustainability problems. Few of the studies reviewed here documented whether co-payments, deductibles, or medical utilization reviews were used in limiting moral hazard. In Rwanda, the national health insurance plan required individuals to pay 10% of treatment costs as co-payment (24); similarly, CBHI in India required individuals to make a small co-payment at the time of hospitalization (31). In contrast, the public health insurance scheme in Peru offered full financial coverage with no requirement to make a co-payment (10). Aside from these examples, the studies largely omitted to provide information on patients’ cost-sharing. No study in this review mentioned utilization reviews. Similarly, if the high rates of C-sections are due to supplier-induced demand, they will also influence the scheme's financial sustainability due to their higher reimbursement rates for providers. While some studies provided information on providers’ reimbursements for C-sections relative to vaginal deliveries, the studies did not discuss the effect of these higher reimbursement rates on the scheme's financial viability.

In summary, most of the studies do not provide sufficient details about the insurance policy, like rules related to co-payments, to help understand the implications of these policies on the scheme's financial sustainability. These are also short-term evaluations and, therefore, have not yet been able to demonstrate whether the schemes can continue to meet their financial obligations in the long-term.

It is worth noting that the provision of health insurance can lead to higher total expenditure on MH services as a result of increased demand (including increased demand for higher quality of services). A number of studies in the United States have assessed the effect of health insurance on healthcare costs. Results from the Rand Health Insurance Experiment found that health insurance and its effect on demand for health services could only account for part of the rise in health expenditure during 1950 to 1984, and most of the rise is likely due to technological innovation in healthcare (47). However, another study, assessing the market-wide impact of the introduction of Medicare, estimates that half of the rise in healthcare spending in the United States could be attributed to this insurance scheme (48). Evidence from China's social health insurance plans found that cost escalation was largely attributed to changes in hospital financing and physicians’ payment policies and that demand-side interventions (such as co-payments) did not mitigate the rise in costs (49). In Taiwan, following the introduction of the National Health Insurance scheme, total health expenditure initially increased due to increased demand but then grew more slowly because of cost-sharing with patients and certain direct savings from a single payer system (50). The studies included in this review did not provide evidence about the effect of health insurance on the costs of MH services; this review was specifically focused on evaluating the effect of health insurance on the use of MH services and their quality. However, the effect of health insurance on the cost of these services is an important and related component, which has implications on the societal costs of providing health insurance and, therefore, on the long-term sustainability of such schemes.

## DISCUSSION

### Key findings

Many of the studies reviewed here focused on the relationship between health insurance and MH service-use; these studies demonstrated relatively consistent evidence of a positive correlation between health insurance and the use of MH services, except for a few studies which failed to identify a detectable effect. The evidence spanned different geographic locations and different types of insurance. However, only a few of these studies used rigorous methods to identify a causal effect. Of these, only one study used PSM and included health status as a matching variable; yet, this study finds no detectable effect of health insurance on the use of MH services.

A number of studies presented suggestive evidence that insurance contributed to overprovision of C-sections. Most of the evidence was from Latin American countries and China with examples from different insurance schemes, although one study focused on DR Congo. The studies did not conclusively demonstrate the presence of supplier-induced demand, given that only one study used rigorous methods, and these studies did not demonstrate whether the sample had a previously unmet need for C-sections.

Few studies focused on the relationship between health insurance and the quality of MH services. These studies provided inconclusive evidence because they used different methods for measuring quality and found different directional effects. While there was little evidence on the relationship between health insurance and maternal and neonatal health outcomes, the available evidence was also contradictory. Only two studies assessed the effect of insurance on maternal mortality; only one was rigorously conducted, and it failed to identify an effect. Certain studies found that insurance was positively correlated with lower neonatal mortality; however, there were methodological concerns with these studies which limit causal inference. The absence of detectable effects from insurance on maternal and neonatal mortality may partly be due to sample-sizes that were insufficient to identify such effects. Studies highlighted contextual factors which may limit the effectiveness of insurance; these factors include geographic barriers and the quality of healthcare providers.

### Recommendations

In comparison with the available evidence on other financial incentives and MH outcomes, there appears to be more robust and consistent evidence regarding health insurance. Nonetheless, there are still areas in which further research would be beneficial. The following are the recommendations for future work on research methods, research questions, and tools relating to insurance and MH services and maternal and neonatal outcomes.

#### Research methods

The evidence regarding the relationship between health insurance and the use of MH services is relatively consistent across studies which used different rigorous research methodologies. While many of these studies used rigorous methods, such as PSM, there is still concern that the positive correlation between health insurance and the use of MH service is not causal (or not entirely causal) but represents potential selection effects relating to insurance uptake. The direction of the relationship is consistent with evidence from the RAND randomized controlled study in the United States, demonstrating the positive impact of health insurance on the use of health services in general (51) as well as evidence from a randomized study in Nicaragua, establishing the impact of health insurance on the use of health services in general (52). No randomized controlled trial has been conducted in a low-income country, focusing specifically on health insurance and the use of MH services; such research could provide conclusive, causal evidence which would corroborate the findings from this review and demonstrate the extent to which the effect of health insurance on the use of MH services is attributed to health insurance itself versus other factors, such as income and health status. In addition, the inconsistency in the evidence on the relationship between health insurance and the quality of MH services as well as maternal and neonatal health outcomes suggests the need for additional studies in these areas. Other rigorous methods, such as DD, IV, and PSM (using comprehensive matching variables), could provide such evidence.

The review also identified potential overprovision of C-sections associated with insurance. However, the role that health insurance may play in influencing this overprovision has not been conclusively demonstrated. Further efforts in identifying the presence of supplier-induced demand is necessary. Such research would complement ongoing efforts led by a working group of the Child Health Epidemiology Reference Group and the Maternal Health Task Force to improve measurements of under- and overprovision of C-sections by developing new indicators that identify when decisions about C-sections are being made and by whom (provider or woman), proposing different prospective and retrospective data-collection efforts, and expanding the application of the Robson classification of C-sections to low-income country settings, by using this facility-based classification system to categorize C-sections by women's level of risk (53).

Opportunities should also be sought to use larger sample-sizes to address questions regarding impact of health insurance on maternal and neonatal health outcomes. Cross-country analyses (using pooled DHS data, for example) could be used in identifying such effects. There is evidence on the effect of insurance on the use of MH services as well as evidence on the effectiveness of these services in reducing adverse maternal and neonatal outcomes. However, a rigorous study combining this evidence would conclusively demonstrate the health impacts of insurance.

#### Research areas

The effect of health insurance on the use of MH services will be closely tied with the extent to which the benefits package comprehensively covers ANC, PNC, and delivery-related services. Most of the studies that were reviewed provided information about the MH-related services covered by the insurance product, thereby making it possible to compare whether the inclusion of these specific MH services in the benefits package is related to the use of those particular MH services. Nonetheless, there are some examples of studies which assessed the effect of health insurance on the use of ANC but highlighted that coverage of ANC services varies by county or district (32,33). Future research on the effect of health insurance related to MH services should include specific details regarding the MH benefits package, to make it possible to compare whether their inclusion in the benefits influences their use. Differences in the benefits package, such as insurance that covers only emergency obstetric care versus insurance that comprehensively covers delivery-related costs as well as ANC and PNC, may differentially affect healthcare-seeking behaviours for insured pregnant women. Evaluations assessing how differences in the MH benefits package influences the use and provision of MH services can inform how best to structure MH insurance coverage.

There are many other components of an insurance programme, including the co-payments, waiting period, and provider payments, each of which can incentivize different behaviours both for enrollees and providers. There would be benefit to understanding the differential effects of each of these components. Evaluating the effect of different payment policies on the rate of C-sections can inform the extent to which these compensation mechanisms influence C-section rates and, therefore, inform the suitable structure for provider payments. Given the potentially important role that geographic barriers may play in limiting the effectiveness of health insurance to expand access to care, research on the effect of transportation vouchers included as part of insurance packages could help identify the extent to which geographic constraints play a role in influencing the impact of insurance policies in reducing barriers to access.

The review also identified inconsistencies in the way that quality of MH services was measured, making it difficult to compare findings of the studies. A more comprehensive and consistent methodology for measuring the quality of MH services would help ensure that studies assessing this outcome are capturing meaningful measures of quality. Additional studies could also inform the extent to which the effect of insurance on quality influences maternal and neonatal health outcomes.

Many of the reviewed studies represented evaluations that are conducted a few years after the introduction of the insurance scheme. Longer-term evaluations would also be beneficial, given that there may be concerns regarding the sustainability of these schemes. In addition, while this review was not specifically focused on assessing the effect of health insurance on the cost of MH services, this is an important and related issue which influences the sustainability of these insurance programmes. While there is evidence on the effect of health insurance on healthcare costs in general, the evidence is conflicting (depending on the context), and there is limited evidence relating to MH services in particular.

This review focused on whether health insurance affects the use and quality of MH services. Financial risk protection was not evaluated in this review but is worthy of further study. Many of the papers provided evidence relating to how insurance affects individuals’ out-of-pocket payments. Other spending decisions, such as on schooling, housing, and food, may be affected by households facing unexpected health shocks, such as a delivery complication. These effects on household spending represent other effects from insurance that could be taken into account in considering the broader impact of insurance coverage in relation to pregnant women and mothers.

While this review focused specifically on health insurance, other financial incentives, such as the removal of user fees, can also influence the use and provision of MH services. The evidence reviewed on user fee exemptions also found a consistent positive relationship between user fee removal and facility-based deliveries, for example. Direct comparisons between the effect of free care versus health insurance is difficult, given the different contexts and populations targeted in the different studies. Nonetheless, the evidence from the *Yeshasvini* scheme, in Karnataka, India, highlighted that these different financial incentives can also interact with each other. In this example, insurance did not necessarily remove cost barriers but instead provided access to higher-quality providers. Further study on the comparative effect of different financial incentives could be beneficial.

Since the external validity of these studies depends on being able to apply findings of a study to a similar context, there is a continual need for replication of studies in different countries, focusing on different types of insurance schemes and populations with different health needs.

#### Tools and guidance

In the absence of rigorous evidence on the viability and sustainability of health insurance schemes, particularly for micro-health insurance and CBHI and especially those that provide maternity benefits, implementation research can help inform product design, educational resources, and healthcare delivery. Increasing efforts are being made to capture these lessons from practitioners and to share the knowledge with other key stakeholders. One example is an inventory being developed by the Microinsurance Network on emerging lessons learnt. The publicly available inventory seeks to consolidate product details on both active and inactive health insurance schemes and document successes and failures in design, administration, and delivery of health insurance products and healthcare services.

Another tool that would be useful for researchers and policy-makers is a standardized method for assessing the quality of MH services. Such a tool would enable researchers to provide evidence that can be more easily compared across studies. In addition, this tool could be used in identifying which quality indicators are associated with better maternal and neonatal health outcomes and which are most cost-effective. Insurance policies could use this evidence to target the specific quality indicators as part of their provider reimbursement schemes.

#### Policy

The evidence suggesting that health insurance increases the use of MH services provides justification for promoting broad access to health insurance coverage. In particular, health insurance should offer a comprehensive package of MH services, including ANC, intrapartum and immediate postnatal care plus emergency obstetric care, to affect both use of MH services and potentially maternal health outcomes. Given the findings from this review, it is also important that the package be designed to specifically address the health needs of the target population. For example, payments to providers should be adjusted to avoid the over-provision of services, such as C-sections in contexts where this is a concern. However, in low-income contexts where women have limited access to emergency obstetric care, the underprovision of these services means that the incentives should be structured differently.

Finally, policy-makers, donors, and other relevant stakeholders should consider how insurance and maternal health services are tied into the broader discussion around universal health coverage. The potential comprehensiveness of insurance coverage should encourage policy-makers to address health services in an integrated, interconnected fashion rather than through the lens of disease-specific silos. The policy debate around universal health coverage has relevance for the maternal health community, just as these MH findings have relevance for the broader discussion around health insurance and universal health coverage.

## ACKNOWLEDGEMENTS

We thank Dr. Marjorie Koblinsky and Dr. Mary Ellen Stanton at the United States Agency for International Development (USAID), Dr. Marty Makinen at Results for Development, and Dr. Thierry van Bastelaer at Abt Associates Inc. for their invaluable feedback and guidance throughout the study. We would also like to acknowledge funding received from the Health Finance and Governance Project, Cooperative Agreement # AID-OAA-A-12-00080, funded by the United States Agency for International Development (USAID).

## References

[1] World Health Organization (2012). Trends in maternal mortality: 1990 to 2010. WHO, UNICEF, UNFPA and the World Bank estimates.

[2] Rajaratnam JK, Marcus JR, Flaxman AD, Wang H, Levin-Rector A, Dwyer L (2010). Neonatal, postneonatal, childhood, and under-5 mortality for 187 countries, 1970-2010: a systematic analysis of progress towards Millennium Development Goal 4. Lancet.

[3] United Nations The Millennium Development Goals report 2012. New York, NY: United Nations, 2012. 68 p.

[4] Campbell OMR, Graham WJ, Lancet Maternal Survival Series Steering Group (2006). Strategies for reducing maternal mortality: getting on with what works. Lancet.

[5] Bulatao RA, Ross JA (2003). Which health services reduce maternal mortality? Evidence from ratings of maternal health services. Trop Med Int Health.

[6] Knippenberg R, Lawn JE, Darmstadt GL, Begkoyian G, Fogstad H, Walelign N (2005). Lancet Neonatal Survival Steering Team. Systematic scaling up of neonatal care in countries. Lancet.

[7] Cutler DM Zeckhauser RJ The anatomy of health insurance. *In*: Culyer AJ, Newhouse JP, editors. Handbook of health economics. 1st ed. Oxford: Elsevier, 2011:563-643.

[8] Wang H, Switlick K, Ortiz C, Zurita B, Connor C (2012). Health insurance handbook: how to make it work.

[9] Wagstaff A, Lindelow M, Jun G, Ling X, Juncheng Q (2009). Extending health insurance to the rural population: an impact evaluation of China's new cooperative medical scheme. J Health Econ.

[10] Bitran R Muñoz R Prieto L Health insurance and access to health services, health services use, and health status in Peru. *In*: Escobar M-L, Griffin CC, Shaw RP, editors. The impact of health insurance in low- and middle-income countries. Washington, DC: Brookings Institution Press, 2010:106-21.

[11] Srivastava S, Majumdar A, Singh JR (2010). Financial inclusion opportunities for micro health insurance in Nepal: an exploratory analysis of health incidence, costs and willingness to pay in Dhading and Banke Districts of Nepal.

[12] Churchill C Dalal A Ling J Pathways towards greater impact: better microinsurance models, products and processes for MFIs. Geneva: International Labour Organization, 2012. 47 p. (Microinsurance paper no. 18).

[13] Aggarwal A (2010). Impact evaluation of India's ‘Yeshasvini’ community-based health insurance programme. Health Econ.

[14] Chen Y, Jin GZ (2012). Does health insurance coverage lead to better health and educational outcomes? Evidence from rural China. J Health Econ.

[15] Mensah J, Oppong JR, Schmidt CM (2010). Ghana's national health insurance scheme in the context of the health MDGs: an empirical evaluation using propensity score matching. Health Econ.

[16] Lu C, Chin B, Lewandowski JL, Basinga P, Hirschhorn LR, Hill K (2012). Towards universal health coverage: an evaluation of Rwanda Mutuelles in its first eight years. PLoS One.

[17] Giedion U, Florez CE, Diaz BY, Alfonso E, Pardo R, Villar M, Escobar M-L, Griffin CC, Shaw RP (2010). Columbia's big bang health insurance reform. The impact of health insurance in low- and middle-income countries.

[18] Rosenbaum PR, Rubin DB (1983). The central role of the propensity score in observational studies for causal effects. Biometrika.

[19] Kozhimannil KB, Valera MR, Adams AS, Ross-Degnan D (2009). The population-level impacts of a national health insurance program and franchise midwife clinics on achievement of prenatal and delivery care standards in the Philippines. Health Policy.

[20] McQuestion MJ, Velasquez A (2006). Evaluating program effects on institutional delivery in Peru. Health Policy.

[21] Cai W-W, Marks JS, Chen CHC, Zhuang Y-X, Morris L, Harris JR (1998). Increased cesarean section rates and emerging patterns of health insurance in Shanghai, China. Am J Public Health.

[22] Celik Y, Hotchkiss DR (2000). The socio-economic determinants of maternal health care utilization in Turkey. Soc Sci Med.

[23] Chankova S, Atim C, Hatt L, Escobar M-L, Griffin CC, Shaw RP (2010). Ghana's national health insurance scheme. The impact of health insurance in low- and middle-income countries.

[24] Hong R, Ayad M, Ngabo F (2011). Being insured improves safe delivery practices in Rwanda. J Community Health.

[25] Sekabaraga C, Diop F, Soucat A (2011). Can innovative health financing policies increase access to MDG-related services? Evidence from Rwanda. Health Policy Plan.

[26] Smith KV, Sulzbach S (2008). Community-based health insurance and access to maternal health services: evidence from three West African countries. Soc Sci Med.

[27] Victora CG, Matijasevich A, Silveira MF, Santos IS, Barros AJD, Barros FC (2010). Socio-economic and ethnic group inequities in antenatal care quality in the public and private sector in Brazil. Health Policy Plan.

[28] Cercone J, Pinder E, Jimenez JP, Briceno R, Escobar M-L, Griffin CC, Shaw RP (2010). Impact of health insurance on access, use, and health status in Costa Rica. The impact of health insurance in low- and middle-income countries.

[29] Barros FC, Victora CG, Barros AJ, Santos IS, Albernaz E, Matijasevich A (2005). The challenge of reducing neonatal mortality in middleincome countries: ﬁndings from three Brazilian birth cohorts in 1982, 1993, and 2004. Lancet.

[30] Bogg L, Wang K, Diwan V (2002). Chinese maternal health in adjustment: claim for life. Reprod Health Matters.

[31] Devadasan N, Criel B, Van Damme W, Manoharan S, Sarma PS, Van der Stuyft P (2010). Community health insurance in Gudalur, India, increases access to hospital care. Health Policy Plan.

[32] Long Q, Zhang T, Hemminki E, Tang X, Huang K, Xiao S (2010). Utilisation, contents and costs of prenatal care under a rural health insurance (New Co-operative Medical System) in rural China: lessons from implementation. BMC Health Serv Res.

[33] Schneider P, Diop F (2001). Impact of prepapyment pilot on health care utilization and financing in Rwanda: findings from final household survey.

[34] Adinma ED, Brian-D Adinma JI, Obionu CC, Asuzu MC (2011). Effect of government-community healthcare co-financing on maternal and child healthcare in Nigeria. West Afr J Med.

[35] Adinma ED, Nwakoby BA, Adinma BD (2010). Integrating maternal health services into a health insurance scheme: effect on healthcare delivery. *Nig Q J Hosp*. Med.

[36] Renaudin P, Prual A, Vangeenderhuysen C, Ould Abdelkader M, Ould Mohamed Vall M, Ould El Joud D (2007). Ensuring financial access to emergency obstetric care: three years of experience with Obstetric Risk Insurance in Nouakchott, Mauritania. Int J Gynaecol Obstet.

[37] Huntington D, Banzon E, Recidoro ZD (2012). A systems approach to improving maternal health in the Philippines. Bull World Health Organ.

[38] Zweifel P, Manning WG, Culyer AJ, Newhouse JP (2001). Moral hazard and consumer incentives in health care. Handbook of health economics.

[39] McGuire TG, Culyer AJ, Newhouse JP (2001). Physician agency. Handbook of health economics.

[40] Murray SF, Serani Pradenas F (1997). Cesarean birth trends in Chile, 1986 to 1994. Birth.

[41] Murray SF (2000). Relation between private health insurance and high rates of caesarean section in Chile: qualitative and quantitative study. BMJ.

[42] Bogg L, Huang K, Long Q, Shen Y, Hemminki E (2010). Dramatic increase of cesarean deliveries in the midst of health reforms in rural China. Soc Sci Med.

[43] Moore B (1985). Appropriate technology for birth. Lancet.

[44] Stanton CK, Holtz SA (2006). Levels and trends in cesarean birth in the developing world. Stud Fam Plann.

[45] Gibbons L, Belizan JM, Lauer JA, Betran AP, Merialdi M, Althabe F (2012). Inequities in the use of cesarean section deliveries in the world. Am J Obstet Gynecol.

[46] Criel B, Van der Stuyft P, Van Lerberghe W (1999). The Bwamanda hospital insurance scheme: effective for whom? A study of its impact on hospital utilization patterns. Soc Sci Med.

[47] Manning WG, Newhouse JP, Duan N, Keeler EB, Leibowitz A, Marquis MS (1987). Health insurance and the demand for medical care: evidence from a randomized experiment. Am Econ Rev.

[48] Finkelstein A (2007). The aggregate effects of health insurance: evidence from the introduction of Medicare. Quar J Econ.

[49] Liu X, Hsiao WCL (1995). The cost escalation of social health insurance plans in China: its implication for public policy. Soc Sci Med.

[50] Lu J-FR, Hsiao W (2003). Does universal health insurance make health care unaffordable? Lessons from Taiwan. Health Aff (Millwood).

[51] Newhouse JP, Insurance Experiment Group (1993). Free for all? Lessons from the RAND health insurance experiment.

[52] Thornton RL, Hatt LE, Field EM, Islam M, Diaz FS, González MA (2010). Social security health insurance for the informal sector in Nicaragua: a randomized evaluation. Health Econ.

[53] Robson M (2001). Classification of caesarean sections. Fetal Matern Med Rev.

